# Design and Validation of a Modular One-To-Many Actuator for a Soft Wearable Exosuit

**DOI:** 10.3389/fnbot.2019.00039

**Published:** 2019-06-18

**Authors:** Michele Xiloyannis, Eugenio Annese, Marco Canesi, Anil Kodiyan, Antonio Bicchi, Silvestro Micera, Arash Ajoudani, Lorenzo Masia

**Affiliations:** ^1^Robotics Research Centre, Interdisciplinary Graduate School, Nanyang Technological University, Singapore, Singapore; ^2^Sensory-Motor Systems Lab, Department of Mechanical and Process Engineering, Institute of Robotics and Intelligent Systems, ETH Zürich, Zurich, Switzerland; ^3^Moveo Walks, Inc., Cambridge, MA, United States; ^4^Egicon S.R.L., Modena, Italy; ^5^Gait Up S.A., Lausanne, Switzerland; ^6^SoftRobotics Lab for Human Cooperation and Rehabilitation, Istituto Italiano di Tecnologia, Genoa, Italy; ^7^Department of Information Engineering, Research Center “E. Piaggio”, Università di Pisa, Pisa, Italy; ^8^Bertarelli Foundation Chair in Translational Neuroengineering, Center for Neuroprosthetics and Institute of Bioengineering, École Polytechnique Fédérale de Lausanne, Lausanne, Switzerland; ^9^Director of the Neuro-X Center, Head of Translational Neural Engineering Area, The BioRobotics Institute Scuola Superiore Sant'Anna, Pisa, Italy; ^10^Human-Robot Interfaces and Physical Interaction Lab, Istituto Italiano di Tecnologia, Genoa, Italy; ^11^Institut für Technische Informatik (ZITI), Heidelberg University, Heidelberg, Germany

**Keywords:** soft exosuit, underactuation, assistive robots, unidrive, soft robotics, exoskeletons, PWM control, wearable robotic suit

## Abstract

The size, weight, and power consumption of soft wearable robots rapidly scale with their number of active degrees of freedom. While various underactuation strategies have been proposed, most of them impose hard constrains on the kinetics and kinematics of the device. Here we propose a paradigm to independently control multiple degrees of freedom using a set of modular components, all tapping power from a single motor. Each module consists of three electromagnetic clutches, controlled to convert a constant unidirectional motion in an arbitrary output trajectory. We detail the design and functioning principle of each module and propose an approach to control the velocity and position of its output. The device is characterized in free space and under loading conditions. Finally, we test the performance of the proposed actuation scheme to drive a soft exosuit for the elbow joint, comparing it with the performance obtained using a traditional DC motor and an unpowered-exosuit condition. The exosuit powered by our novel scheme reduces the biological torque required to move by an average of 46.2%, compared to the unpowered condition, but negatively affects movement smoothness. When compared to a DC motor, using the our paradigm slightly deteriorates performance. Despite the technical limitations of the current design, the method proposed in this paper is a promising way to design more portable wearable robots.

## 1. Introduction

One of the earliest attempts to develop a wearable robotic device to assist human motion, dating back to 1967, failed because of the excessive weight and size of the system (Mosher, 1967). Since then, advancements in material science, power supplies and computing power have fundamentally broadened the boundaries of what we can achieve.

Exoskeletons have been used for a plethora of applications, ranging from performance augmentation in industry (de Looze et al., 2016) to neuro-rehabilitation in medicine (Louie and Eng, 2016). Despite exciting achievements, there are still substantial technical limitations preventing wearable powered devices from becoming a ubiquitous part of our daily lives. Among others, power requirements and weight of the actuation stage play a key role, confining most of the existing exoskeletons to research laboratories or specialized clinics.

A significant step forwards in this direction has been recently taken by using fabric and polymers to transmit forces and torques to the human body. Soft materials limit the magnitude and accuracy of assistive forces but allow to engineer lighter, less power-demanding, and svelter exoskeletons, resembling our everyday clothes more than the rigid machines portrayed by science-fiction movies (Asbeck et al., 2014).

Although fundamental research is being carried out to design efficient, controllable and robust new actuators (Cappello et al., 2018), most exosuits are powered by traditional electric motors, transferring power to the joints through flexible transmissions (Asbeck et al., 2013). Using one motor to assist each Degrees of Freedom (DoF) of the human body is the most common design choice. This strategy is hardly scalable to complex systems: the human arm alone has at least 7 DoFs and the complexity, size, and weight of a device using such a high number of motors would make it impractical.

**Figure 1:**
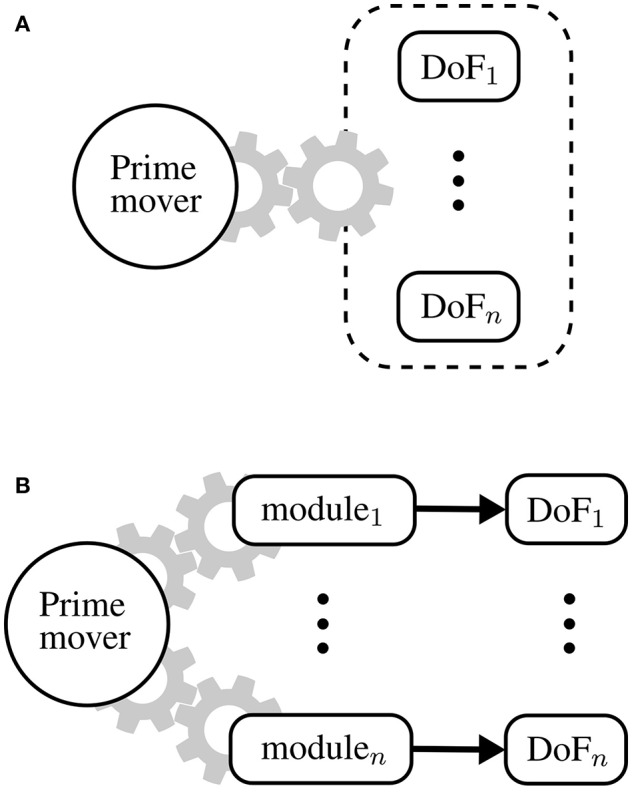
Underactuation mechanisms and One-To-Many (OTM) paradigm. **(A)** Typical underactuated mechanisms rely on some form of mechanical coupling between degrees of freedom (DoF), imposing a hard constraint on the kinematics and/or dynamics of the system. **(B)** The OTM paradigm consists in actuating many DoF, each tapping power from the same drive but controlled independently by a local module.

A common way to address this problem is to use fewer motors than DoF: underactuation strategies include differential mechanism (In and Cho, 2015), mechanical implementation of kinematic synergies (Catalano et al., 2014; Xiloyannis et al., 2016) and routing of the driving cables along multiple joints (Asbeck et al., 2015). However, these approaches impose hard constrains on the kinetics and/or kinematics of the wearer, allowing only a finite number of predefined moving patterns. This idea is conceptually shown in Figure 1A, where multiple DoF are mechanically coupled to be driven by a single motor.

An interesting, yet less investigated, method involves using a set of modules, each one moving a DoFs, all tapping mechanical energy from a single drive. In literature, this paradigm is known as One-To-Many (OTM) (Hunt et al., 2013), Unidrive (Karbasi et al., 2004), or Single-Motor-Driven (SMD) system (Chen and Xie, 1999).

The idea of having a prime mover delivering motion to many subunits is gracefully illustrated in Dante Alighieri's picture of the structure of the universe: he imagined the existence of an outer rotating spheric “sky” that generates energy and transfers it trough its motion to its inner circles, each rotating at a fraction of its speed:

“*Non è suo moto per altro distinto*,

*ma li altri son mensurati da questo*,

*sì come diece da mezzo e da quinto.”*^1^

A One-To-Many transmission is the mechanical equivalent of this idea: one electric motor, that we shall call prime mover, transfers power to many modules, each driving a DoF. This paradigm is shown in Figure 1B. The prime mover rotates at a constant speed and the trajectory of each DoF is modulated locally by its corresponding module. Such a setup, unlike underactuation mechanisms, allows independent control of each DoF and scales nicely with increasingly complex systems.

The challenge when designing an OTM system comes down to engineering a module that is smaller and less power-consuming than a motor but can control the position, velocity, and/or torque of a joint just as well.

Performance-wise, Infinite Variable Transmissions (IVT), allowing to continuously modulate their transmission ratio within a range of positive and negative values, are the ideal candidates for an OTM module. The input velocity remains constant while the output velocity of each DoF can be modulated and even reversed by changing the transmission ratio of each IVT. The recently-published work from Kembaum et al. proposed a novel compact design (Kembaum et al., 2017), but traditionally, the size and mass of IVTs would not justify their use over a simple additional motor. Hunt et al. proposed an OTM system where each module consisted of a linear spring and a clutchable ratchet, the former used to store energy and the latter to selectively release it (Hunt et al., 2013).

The group led by Kermani (Kermani and Alex, 2014; Yadmellat et al., 2014), proposed a mechanism employing magnetorheological (MR) clutches to design a 2 DoF manipulator for safe human-robot interaction. A single electric motor was placed a the base of the robot, providing power to both joints, while three MR clutches were controlled to limit the output torque's magnitude and direction at each joint. The authors showed that this Distributed Active-Semi Active (DASA) actuation paradigm can achieve smooth and accurate tracking of joint positions while benefiting from key advantages of MR clutches such as backdrivability and low impedance.

Finally, a handful of research groups have presented designs based on a similar and powerful principle: the module consists of at least two gears, constantly rotating in opposite directions, and the output is coupled with either one of them using ElectroMagnetic (EM) clutches, or locked using a brake (Li et al., 2003; Karbasi et al., 2004; Yadmellat et al., 2014). The group led by Xie was probably one of the first to propose such arrangement to drive a 9 DoF robotic hand (Chen and Xie, 1999) and a 6 DoF serial manipulator (Li et al., 2011) with a single electric motor.

**Figure 2:**
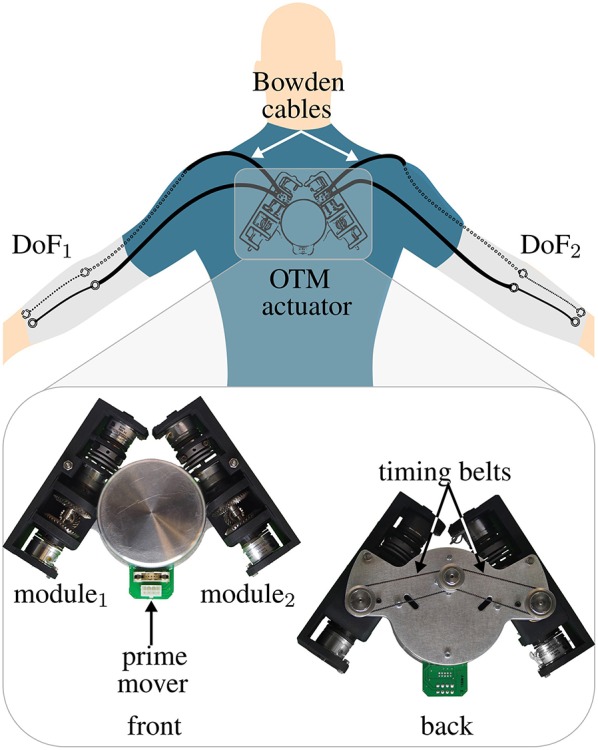
OTM actuator to power two elbow exosuits with one electric motor. This system, that we proposed in Canesi et al. (2017), consists in one prime mover, transmitting power to two clutchable DOF Modules connected to two elbow exosuits via Bowden cables. Each module independently controls the position of its corresponding DoF.

The encouraging results of OTM systems, applied to drive manipulators and robotic hands, led us to investigate their feasibility in the field of wearable assistive devices, increasingly in need of novel, efficient actuation paradigms. We previously proposed an OTM system, consisting of 2 clutchable modules, to actuate the tendons driving a soft exosuit for the elbow joints (Canesi et al., 2017), shown in Figure 2. Each module followed a working principle similar to the one presented in Chen and Xie (1999): this design has the advantage, over the spring-ratchet design in Hunt et al. (2013), of not being limited in the amount of energy that can be stored in each module and it results in a simpler and lighter architecture than the designs employing MR clutches or IVTs.

Our first work was controlled using a heuristically-designed state machine that didn't guarantee stability. Because of this lack of robustness we limited our first assessments to a testbench. Even with a simple controller, however, we demonstrated the feasibility of this approach to achieve independent control of multiple DoF, using a single drive. In this manuscript, we bring the assessment one step further, by including the human in the validation process.

We propose a refined version of our module's design and presents a novel PID-modulated PWM controller to finely adjust the velocity of each DoF independently. We thoroughly characterize the system on a test-bench and then use it as the low-level layer of an admittance-based scheme to control our soft exosuit.

The performance of the new actuation unit is finally compared to that of a traditional DC motor by assessing their impact on kinetics and kinematics of human movement. This human-in-the-loop validation highlights the limitations of our approach and points out avenues for improvement.

## 2. OTM Design and Control

**Figure 3:**
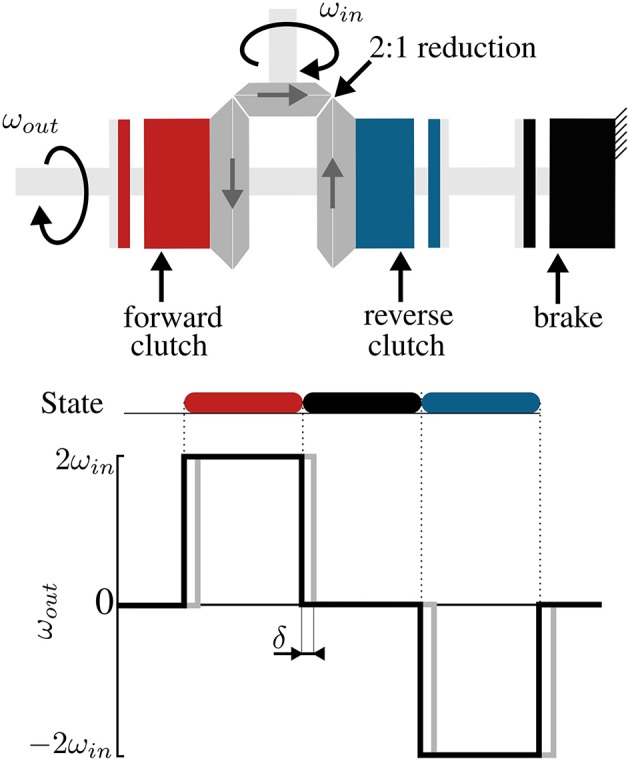
Working principle of a DOF module. ElectroMagnetic (EM) clutches are used to convert a unidirectional input to a bidirectional output by coupling an output shaft with either one of two counter-rotating gears; a brake locks the output in place. Black lines in the plot show the ideal behavior of the clutches, gray lines the actual one, caused by a delay in engagement and disengagement of the armature and rotor.

The working principle of the clutchable OTM module is shown in Figure 3. The device consists of three EM clutches (SO11, Inertia Dynamics, 5 W) used for coupling the output to either a forward-rotating gear (red), a reverse-rotating gear (blue), or to lock it (black). Depending on which clutch is engaged, the module works in four possible states:

– Free: when all the clutches are disengaged, the output velocity at the pulley is undefined, the output is back-drivable.
– Lock: when the brake is engaged.
– Forward: when the “forward” clutch is engaged, the output velocity equals the input velocity times the reduction ratio between the input and output ports.
– Reverse: when the “reverse” clutch is engaged, the output velocity equals the reverse of the input velocity times the reduction ratio between the input and output ports.

Table 1 summarizes the states of the module, where the engagement of each clutch is represented by a binary variable. Any other pattern of activation is avoided as it would result in mechanical stall of the prime mover.

**Table 1:** Possible states of the module.

**State**	**Forward clutch**	**Reverse clutch**	**Brake**
Free	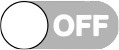	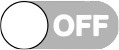	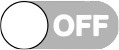
Forward	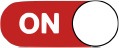	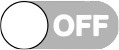	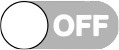
Reverse	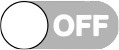	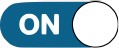	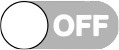
Lock	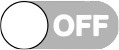	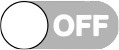	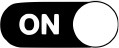

### 2.1. Mechanical Design

**Figure 4:**
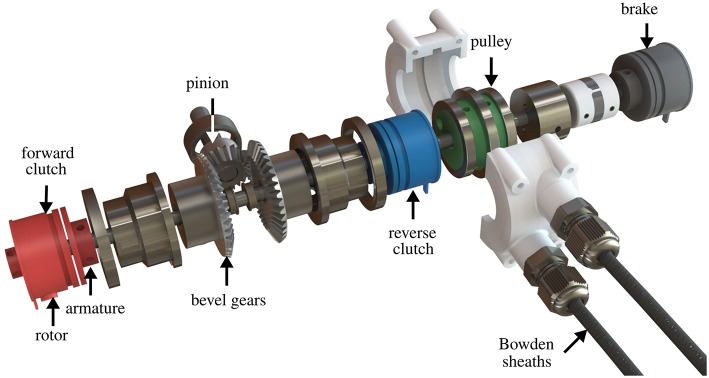
Mechanical design of a One-To-Many (OTM) module. Each module receives power from the prime mover through a pinion, connected to two, opposite-facing, bevel gears that rotate in opposite directions. Each bevel gear can be coupled to the passing countershaft by engaging an ElectroMagnetic (EM) clutch, selectively rotating it in either direction. A third EM clutch can connect the countershaft to the frame, acting as a break. The shaft drives a pulley housing the antagonistic Bowden cables that actuate the soft exosuit.

**Figure 5:**
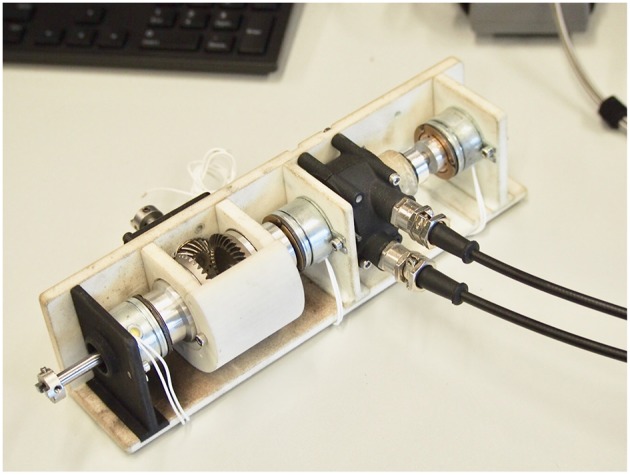
A single DoF assembled Module.

Figure 4 shows an exploded view of the functional components of the module. A pinion is continuously rotated by the prime mover and meshes orthogonally with two bevel gears facing each other. Thanks to this arrangements, the bevel gears rotate in opposite directions. An aluminum link couples each gear to the armature of an EM clutch, whose rotor is rigidly linked to a passing countershaft. When power is applied to either one of the EM clutches, the armature is coupled to the rotor, thus effectively linking the countershaft to either one of the gears. A third, identical clutch, acts as a brake, locking the countershaft in a static position by coupling it with the frame. A flexible coupling joins the countershaft to a pulley (the output of the module); the pulley houses two cables wrapped in opposite directions and is used to transmit motion to the exosuit through flexible Bowden cables. Figure 5 shows a photograph of an assembled module, enclosed in a 3D printed casing.

### 2.2. Control

Figure 3 shows that the module has three working states: forward, reverse, and brake. In Canesi et al. (2017), we proposed a heuristic control approach that consisted in a feedback state machine to keep the measured trajectory as close as possible to a reference profile. This strategy, although being simple, presented fundamental limitations in accuracy and stability.

In this work, we propose a more principled paradigm to control this discrete system, based on the work of Karbasi et al. (2004). The controller is based on Pulse Width Modulation (PWM) of the three discrete states of the OTM, regulated by a feedback PI controller, to continuously adjust the average velocity of the module's output. The non-linear PI controller translates the difference between the desired and actual velocity of the module to a rectangular pulse signal, having value −1 (reverse clutch), 0 (brake), or 1 (forward clutch), whose duration in time is dependent on the magnitude of the error.

In the next sections we describe how a tracking error is converted to a discrete control signal for the clutches for an ideal PWM-regulated system.

Using a state-space representation, we can describe a non-linear PWM-controlled system with the following equations:

$$\begin{array}{l} \{\begin{array}{c} \frac{dx}{dt}=u\left(t\right)\omega_{in}\\ y\left(t\right)=x\left(t\right)\\ e\left(t\right)=r\left(t\right)-y\left(t\right)\\ u\left(t\right)=PWM\left(e\left(t_{k}\right)\right) \end{array} \end{array}$$

where

**Figure 6:**
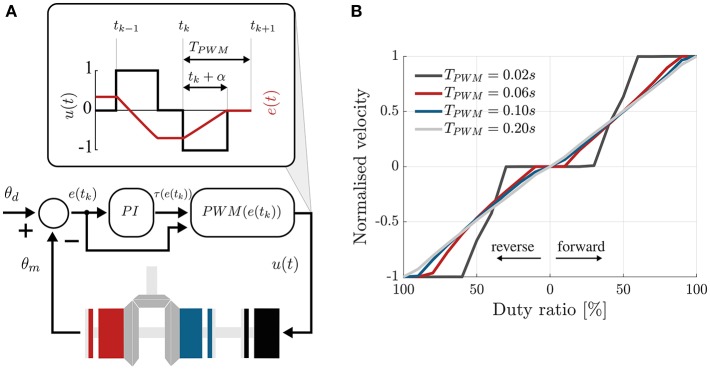
PI-regulated PWM control of the clutchable module. **(A)** The error between the desired and measured position of the countershaft is passed through a traditional PI controller that computes the duty cycle of the control signal, according to Equation (2), in cascade with the PWM function defined in Equation (1). This sets the control signal to 1 or −1 for a fraction of the PWM period that depends on the magnitude of the error. 1 engages the forward clutch, −1 the reverse clutch and 0 the brake. **(B)** Choosing the period of the PWM signal. Velocity of the output shaft, normalized by the input speed, vs. the duty ratio of the PWM signal controlling the forward and reverse clutches. Different colors show different PWM frequencies. For high frequency, low and high duty ratios do not affect the output velocity.

– *u*(*t*) is the control signal for the clutches, bonded to have values −1, 0, or 1.
– *x*(*t*), the state variable; in our case, the angular position of the module's pulley, θ.
– *e*(*t*) is the error between the reference *r*(*t*) and the output *y*(*t*).
– ω_*in*_ is the fixed input velocity of the module.
– *t*_*k*_ represents the instant of initiation of the *k*-th PWM period (Figure 6, top).
– *PWM*(*e*(*t*_*k*_)) is the PWM control operator, defined as:
  $$\begin{array}{l} PWM=\\ \{\begin{array}{cc} \text{sgn}\left(e\left(t_{k}\right)\right) & \;\text{for }t_{k}\leq t\leq t_{k}+\alpha \\ 0 & \;\text{elsewhere} \end{array} \end{array}$$

where we have used α = τ(*e*(*t*_*k*_))*T*_*PWM*_, for simplifying the notation, with τ(*e*(*t*_*k*_)) being the duty ratio function and *T*_*PWM*_ the period of the PWM signal.

The PWM operator basically sets the control signal to 1, 0, or −1 for a period of time defined by τ(*e*(*t*_*k*_)).

This duty ratio function, τ(*e*(*t*_*k*_)), needs to output a value between 0 and 1, representing the ratio for which the control signal will be equal to the sign of the error. Sira-Ramirez suggests the following as a good choice for non-linear systems (Siraramirez, 1989):

(1) $$\tau \left(e\left(t_{k}\right)\right)=\{\begin{array}{cc} \beta |e\left(t_{k}\right)| & \;\text{for}|e\left(t_{k}\right)|\leq 1/\beta \\ 1 & \;\text{for}|e\left(t_{k}\right)|>1/\beta , \end{array}$$

that makes the duty ratio proportional to the magnitude of the error if the error is relatively small (smaller than a threshold 1/β) and saturates it to 1 if the magnitude of the error is larger than the threshold.

The big assumption of this controller is that the clutches behave like ideal switches, i.e., the output velocity of the module instantaneously equals a multiple of the input velocity when the clutch is engaged (Figure 3, black). In practice, this is not the case: the clutches have an intrinsic delay when engaging (shown in gray in Figure 3) and the velocity of the output decays in an exponential-like fashion when the clutch is disengaged (Karbasi et al., 2004).

Skoog and Blankenship (1970) and colleagues have shown that such phenomenon can be mitigated with the addition of an integral feedback term. Adding this to Equation (1) makes the output of the duty ratio function, in the region |*e*(*t*_*k*_)| ≤ 1/β, proportional not only on the magnitude of the error but also to its history, effectively behaving like a non-linear PI controller.

### 2.3. Performance

We characterized the performance of the DOF module for varying control parameters and tested its limits in speed and torque transmission.

Specifically, we evaluated the ability of the controller to modulate the output velocity for varying PMW periods *T*_*PWM*_, we tested its ramp response and bandwidth for varying input velocities of the prime mover and, lastly, its maximum load rating. The DOF module was equipped with an incremental encoder (AMS, AS5047P, 1,000 pulses/rev), monitoring the position of the cable pulley.

#### 2.3.1. Velocity Modulation

Figure 6B shows the ability of the PWM controller to adjust the velocity of the module as its duty cycle changes. The plot shows the normalized velocity (ω_*out*_/ω_*in*_), for varying duty ratio of the PWM signal, where the duty ratio expresses the percentage of time in *T*_*PWM*_, where the control signal is non-zero, i.e., 1 or −1. Lines of different color represent different frequencies of the PWM signal.

The *T*_*PWM*_ should be set as small as possible, to ensure stability, but bigger than the time delay δ, necessary for the clutches to physically engage once powered. Indeed, very high frequencies of the PWM do not result in a higher ability to modulate the output velocity. This is caused by the intrinsic delay, δ, necessary for the rotor and the armature of the EM clutches to engage upon the application of power. We chose *T*_*PWM*_ = 0.20 s, being the highest PWM frequency showing a near-linear trend.

#### 2.3.2. Ramp Response

**Figure 7:**
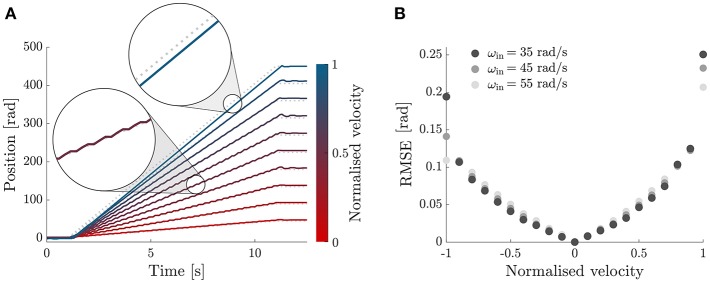
Ramp response. **(A)** Tracking performance of the DOF module with the PI-regulated PWM control. Ramp response for velocities between 10 and 100% of the input velocity. **(B)** RMSE between the desired and measured positions of the module's output, for desired output velocities between -ω_*in*_ and ω_*in*_ (x-axis) and different input velocities (grayscale). The behavior of the controller is symmetric, with increasing error for higher absolute velocities.

Figure 7A shows the tracking ability of our PID-modulated PWM controller to track a ramp position profile of increasing slope, between 10% and 100% of the input velocity, in steps of 10%. The experiment was repeated for three fixed input velocities and showed a consistent behavior, with the tracking ability of the controller deteriorating as the normalized speed approached one.

#### 2.3.3. Bandwidth

**Figure 8:**
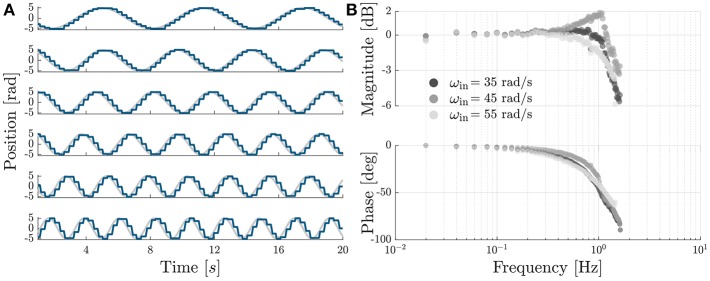
Sinusoidal tracking and Bode plot. **(A)** Sinusoidal tracking accuracy of the OTM module with the PI-regulated PWM control described in section 2.2, shown for six equally-spaced frequencies, between 0.15 and 0.4 Hz. **(B)** Bode plot of the transfer function between desired and measured position of the module's output, shown for three different velocities of the prime mover. The system has a cut-off frequency of 1.26, 1.51, and 1.30 Hz, for a speed of the prime mover of 35, 45, and 55 rad/s, respectively.

Figure 8 shows the tracking of sinusoidal trajectories and Bode plot of the module with the controller proposed in section 2.2. The module was commanded to follow a sinusoidal trajectory of the form:

(2) $$\begin{array}{l} \theta_{out}^{d}\left(t\right)=A\sin\left(2\pi f_{0}t\right) \end{array}$$

with *A* corresponding to the amplitude required for a flexion/extension motion of the elbow of 90 deg and a frequency *f*_0_ evaluated between 0.01 and 1.51 Hz, in incremental steps of 0.02 Hz. For each frequency we collected data for 20 s at a sampling rate of 1 kHz; the first second was discarded for further analysis to evaluate the performance of the system at steady-state. This procedure was repeated for three different input velocities of the prime mover 35, 45, and 55 rad/s.

For each frequency we performed an analysis in the Fourier domain to evaluate the amplitude ratio and the phase lag between the desired *s*(*t*) and the measured θ_*out*_ signal. The *n*th complex coefficient of the Fourier series has the form:

(3) $$\begin{array}{l} C_{n}=\frac{f_{0}}{N}\int_{0}^{\frac{N}{f_{0}}}s\left(t\right)e^{-j2\pi \;nf_{0}t}dt \end{array}$$

where *f*_0_ is the sampling rate and *N* is the number of cycles whereby the signal is repeated. For each driving frequency we evaluated the response as the ratio between the coefficients of the fundamental frequency of the measured and desired signals:

(4) $$\begin{array}{l} H\left(f_{0}\right)=\frac{C_{1}^{\text{measured}}}{C_{1}^{\text{desired}}}. \end{array}$$

Figure 8B shows a Bode plot of the system, representing the transfer function between the measured and desired position of the module. The device and controller show a cut-off frequency of 1.26, 1.51, and 1.30 Hz, for a speed of the prime mover of 35, 45, and 55 rad/s, respectively. We chose a velocity of 45 rad/s for all following tests.

#### 2.3.4. Loaded Behavior

**Figure 9:**
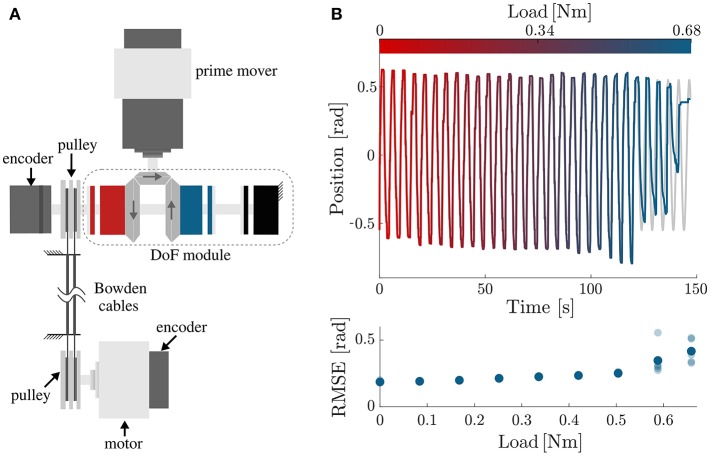
Performance testing under load. **(A)** Testbench used for assessing the performance of the DoF while moving a load. The first motor, i.e., the prime mover, drives the DoF module; the second motor is used to simulate a load on the output. Mechanical power is transmitted from the DoF module to the load via Bowden cables. **(B)** Tracking accuracy of the OTM module, controlled with the PI-regulated PWM controller, under increasing load. The top plot shows the desired (gray) and measured (colored) position of the module's output as the load increases between 0 and 0.68 N m. The clutches start to slip just before 0.6 N m, as quantified by the increase in RMSE shown in the bottom plot.

Finally, we tested the tracking accuracy of the module in the presence of a load. For this testing, we attached the Bowden cables on a second pulley, driven by a DC motor (Maxon EC-i 40, 70 W, 3.7:1 reduction ratio), to apply a load on the end-effector (shown in Figure 9A).

The module was set to follow a sinusoidal trajectory while the load linearly increased between 0 and 0.68 N m (maximum rated torque for the EM clutches). The test was repeated 5 times. Indeed, the clutches started slipping, causing a steep deterioration in tracking accuracy, around 0.6 N m (Figure 9B). This is lower than their rated value, probably due to friction losses in the transmission.

Table 2 summarizes the technical characteristics of our OTM module assembly shown in Figures 4, 5, where the maximum rated torque and velocity have been mapped to the elbow joint.

**Table 2:** Technical specifications.

**Characteristics**	**Values**		
**Module**			
Weight [Kg]	0.534		
Dimensions [cm]	15 × 6 × 5		
	35 rad/s	45 rad/s	55 rad/s
Bandwidth [Hz]	1.26	1.51	1.30
**Assembly**			
DOF	2		
Weight [Kg]	2.2		
Dimensions [cm]	26 × 18 × 12		
Motor power [W]	90		
Max torque^a^ [Nm]	3.4		
Max velocity^a^ [deg/s]	424		

a *At the elbow joint*.

## 3. Testing on Human Movements

To test the feasibility of the OTM mechanism for assistive purposes, we compared the performance of an exosuit driven by the OTM mechanism to that of the exosuit driven by a traditional DC motor. The performance of the device was assessed through its effect on the the kinematics and muscular activity of its wearer.

### 3.1. Exosuit Design and Control

**Figure 10:**
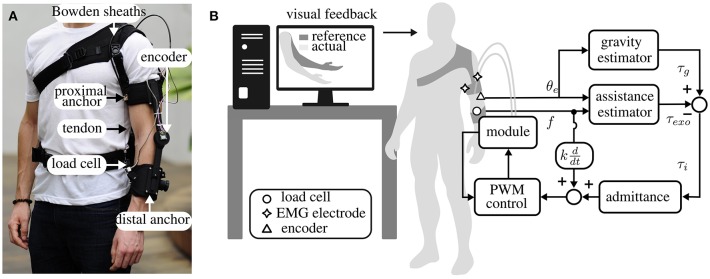
Design and control of the exosuit for the elbow, actuated by a DoF module. **(A)** The suit is driven by Bowden cables that pull together two anchor points on the arm and forearm, generating a torque on the elbow joint. A load cell senses the tension on the cables and an encoder measures the join position. **(B)** Experimental setup and high-level control of the suit. The participant was asked to follow the movement of an avatar on a screen while the suit followed the subject and provided a torque on the elbow equal and opposite to gravity. This was achieved with an admittance controller, using the PWM controller described in section 2.2 as an inner position loop.

The exosuit is shown in Figure 10. The device consists of a frame of soft material that wraps around the arm and forearm and transmits torque to the elbow through artificial tendons. A pair of Bowden cables transmits power from the actuation unit to the joint (Supplementary Material).

The suit is equipped with a force sensor (Futek, LCM300), secured on the distal anchor point, that measures the tension in the flexing tendon, and an absolute encoder (AMS, AS5047P, 1,000 pulses/rev), mounted on a 3D-printed joint between the arm and forearm straps, that monitors the angular position of the joint.

The device provides assistance to its wearer through an admittance-based controller, detailed in Chiaradia et al. (2018). The control paradigm is designed to have the dual purpose of compensating for gravitational forces acting on the forearm and allowing the exosuit to move in concert with its wearer.

A schematic diagram of the controller is shown in Figure 10B. It comprises an outer torque loop and an inner position loop. The former is responsible for tracking the position-dependent torque profile at the elbow, equal and opposite to gravity. A PID-admittance converts the error between the desired and assistive torque at the elbow to a position reference for the actuation stage.

The torque acting on the elbow joint as a result of gravity is estimated using a simple single-joint model and assuming that the arm is adducted on the side of the trunk:

(5) $$\begin{array}{l} \tau_{g}=mgl_{c}\sin\theta_{e}, \end{array}$$

with *m* being the combined mass of the forearm and hand, *l*_*c*_ the distance of the center of gravity of the forearm and hand from the center of rotation of the elbow joint, *g* the acceleration of gravity and θ_*e*_ the elbow angle, assumed to be zero in the fully-extended configuration.

The assistive torque is estimated by multiplying the tension measured by the load cell, *f*, by its moment arm *P*(θ_*e*_) (refer to Xiloyannis et al., 2017 for a full formulation):

(6) $$\begin{array}{l} \tau_{exo}=P\left(\theta_{e}\right)f. \end{array}$$

Using the notation shown in Figure 10B, the difference between τ_*g*_ and τ_*exo*_ is converted to a reference position for the actuation stage, by a specified admittance. The admittance assumes the form of a PID controller (Yu et al., 2011):

(7) $$\begin{array}{l} Y\left(s\right)=\frac{\theta_{e}}{\tau_{g}-\tau_{exo}}=P+\frac{I}{s}+Ds, \end{array}$$

with the P, I, and D constants governing the characteristics of the relation between the interaction force and the exosuit's kinematics. An additional positive feedback term, proportional to the speed of the elbow joint, increases the sensitivity of the device to its wearer's movements.

### 3.2. Experiments and Data Analysis

The aim of this experiment was to compare the effect on human movements of the exosuit driven by an OTM module to that of the exosuit driven by a traditional DC motor. We included a third condition, consisting in unpowered movements, to have a physiological baseline. In all three conditions, we evaluated smoothness and accuracy of movement, biological torque and muscular activation patterns of a healthy subject performing controlled movements of the elbow. The experimental procedure was similar to the one we used in Xiloyannis et al. (2019) to quantify the effects of a soft wearable exosuit on movements of the upper limbs.

The testing was done on one male subject presenting no evidence or known history of skeletal or neurological diseases, and exhibiting intact joint range of motion and muscle strength. At the beginning of each experimental session the participant was informed of the procedure and he signed an informed consent. The procedures, in agreement with the Declaration of Helsinki, was approved by the Institutional Review Board at Nanyang Technological University.

The participant had to follow a reference movement performed by a dummy character on a screen. The position of his own elbow was displayed as a superimposed replica of the reference one to provide visual feedback. In the unpowered condition, the exosuit's tendons were unhooked from the distal anchor point and the motor's power source was turned off.

The reference motion consisted of series of 10 Minimum Jerk Trajectories (MJT), known to correspond well to the movements of healthy subjects (Flash and Hogan, 1985), with amplitude of 80 deg and a peak velocity of 60 deg/s, corresponding to approximally 50% of the average speed in activities of daily living (Buckley et al., 1996).

Raw data from the suit's absolute encoder and load cell was low-pass filtered (second order Butterworth filter, 10 Hz cut-off frequency) and segmented to isolate the 10 movements comprising each condition. The accuracy of movement was quantified by evaluating the Root Mean Square Error (RMSE) between the measured and reference trajectory.

To quantify kinematic smoothness, we used the SPectral ARC length (SPARC) index proposed in Balasubramanian et al. (2015). The SPARC index was estimated on the norm of the elbow's speed.

The measured force on the flexing tendon was mapped to a torque on the joint using Equation (6), this was used as an estimate of the assistive moment delivered by the exosuit, τ_*exo*_. The total torque, τ, required to perform the movement was derived from the inverse dynamics of the human elbow, represented as a simple pendulum using a second order model. The difference between the total and assistive torque,

(8) $$\begin{array}{l} \tau_{bio}=\tau -\tau_{exo}, \end{array}$$

was used to estimate the biological torque exerted by the subject to perform the movement or hold the position.

The output EMG signal of the Delsys Trigno system was processed to extract its linear average envelope using the procedure suggested in Clancy et al. (2002). The Root Mean Square (RMS) of the processed EMG signal was used as an index of the level of activation of a muscle.

### 3.3. Results

**Figure 11:**
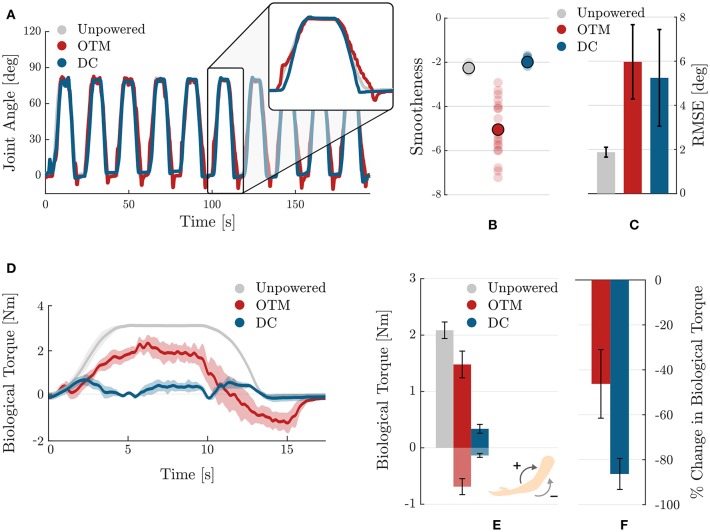
Comparison of the effects of the OTM module and DC motor actuation strategies on the kinetics and kinematics of human movement. **(A)** Trajectories of the elbow when wearing the exosuit in the three tested conditions: unpowered (gray), powered by the OTM module (red), and powered by a traditional DC motor (blue). **(B)** Smoothness of movements as measured by the SPARC coefficient in the three tested conditions. Translucent dots are individual values over the 20 sub-movements of the task, while opaque dots show their mean. **(C)** Accuracy in tracking the reference trajectory, measured by calculating the RMSE, for the three conditions. Movements assisted by the OTM module have similar accuracy to movements assisted by a traditional DC motor. **(D)** Average (solid) and standard deviation across repetitions of the biological torque profiles for the unpowered (gray) condition, for the powered by an OTM module (red) and powered by a traditional DC motor (blue). **(E)** Mean and standard error of the biological torque, averaged across repetitions, for the three tested conditions. **(F)** Change in the overall biological torque required for lifting the arm when assisted by the exosuit. Values are in percentage change of the unpowered case.

Figure 11 compares the characteristics of the elbow's trajectory for the three tested conditions. In Figure 11A, the zoomed-in area shows the overlapping trajectories for the unpowered (gray), powered by an OTM module (red), and powered by a DC (blue) cases: the second condition clearly being more jerky than movements with the DC motor. This is confirmed by a quantitative analysis of the smoothness of movement, evaluated through the SPARC index and shown in Figure 11B.

Figure 11C shows the mean and standard error of the mean of the root mean square error between the reference MJT trajectory and the measured one, in the three conditions. When assisted by the exosuit, whether using the OTM module or a traditional DC motor, movements are less accurate. A smaller difference in mean values exists between the OTM and DC motor cases.

An analysis of the forces transmitted by the exosuit during movement gives further insight on the performance of the OTM module. Figures 11D–F shows the profile and average values of the biological torque, calculated using Equation (8). In the unpowered case, the entire moment required for movement is exerted by the subject; in the OTM case, the positive torque required for movement is reduces, but the exosuit introduces a substantial negative component in the descending phase (Figure 11D).

Figure 11E shows the mean and standard error of the mean, for the positive and negative biological torques exerted in the three conditions. Wearing the exosuit introduces a negative component, required to initiate the downwards motion, both when powered by the DC motor and OTM module. In the latter case, however, the magnitude of negative torque is substantially higher. Overall, the OTM reduces the biological torque at the joint of 46.2%, compared to the unpowered condition, while the DC motor achieves an average −86.3% change.

**Figure 12:**
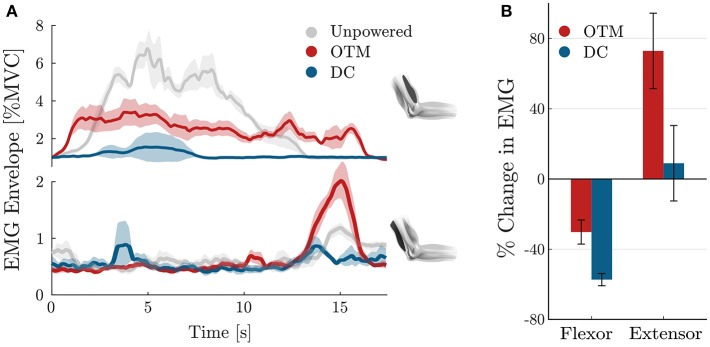
Muscular activation of the biceps brachii and long head of the triceps brachii. **(A)** Average (solid) and standard deviation (shaded) across repetitions of the muscular activation profile during one lifting repetition of the forearm, shown for the unpowered condition (gray), for the exosuit powered by the OTM condition (red), and the exosuit powered by a traditional DC motor (blue). **(B)** Percentage change in the RMS of the muscular activity of the antagonistic muscles, averaged over repetitions.

Finally, Figure 12 analyses the activity of the two major antagonistic muscles involved in delivering power to the elbow joint, namely the biceps brachii and the long head of the triceps brachii. An average and standard deviation of the profile of activation of these muscles is shown in Figure 12A, for the three conditions.

When the suit is unpowered, the biceps brachii reaches a peak activation of 7% of his MVC, while the triceps muscles is relaxed for most of the duration of the movement. When the exosuit, powered by a traditional DC motor, assists its wearer, the activation of the biceps muscles is substantially reduced, with a peak activation during the transient lifting phase. When the device is powered by the OTM module, the agonist muscle's activity is lower than the unpowered condition but presents a slightly different activation pattern, while the triceps shows a peak during the descending phase.

Figure 12B shows the average over repetitions and time, of the RMS of the activity of both muscles for the OTM and DC conditions, expressed in percentage change of the unpowered case. Using a traditional DC motor to actuate the exosuit reduces the activation of the biceps muscle by 57.1% and increases that of the triceps muscle of 8.84%. When the OTM powered the suit, the activation of the biceps muscle was reduced by an average 30% while its antagonist increased by 74%.

## 4. Discussion

Power consumption, weight and size of wearable robots have a fundamental impact on their performance. Soft exosuits, adopting clothing-like materials instead of rigid frames, represent a significant step toward making assistive wearable devices ubiquitous in the near future. Yet, because of the unparalleled complexity of our bodies' biomechanics, we are bounded to either use a high number of motors or underactuation solutions that constrain the natural kinematics of human movements.

The implementation of an actuator that can exploit the torque generated by a single motor to drive multiple DoF would cut down cost, weight and size of the actuation module, reducing the complexity of the control and increasing the overall autonomy of device.

In this study we presented the design and control, and proposed a human-in-the-loop validation of a clutchable modular unit to implement the OTM paradigm for a soft exosuit. The following subsections discuss the implications of our findings first by looking purely at the technical performance of the device and then evaluating its effects on human biomechanics.

### 4.1. On the Performance of the PWM Controller

Figure 6B shows that as the frequency of the PWM controller increases, the output velocity stays null for low duty cycles and saturates to the input velocity earlier, effectively reducing the range of duty cycle where one can modulate the output velocity. This effect is caused by the delay δ, necessary for the armature and rotor of the EM clutches to engage/disengage upon the application of power. Our results are consistent with the observations of Karbasi et al. (2004), who found a similar behavior with wrap-spring clutches: when increasing the PWM frequency, the period *T*_*PWM*_ decreases and, for small duty-ratios, Δ*t* becomes smaller than δ, effectively causing the clutch never to engage. For high duty ratios, on the other hand, the disengagement time, *T*_*PWM*_ − Δ*t* is smaller than the minimum time required for the clutch to disengage, causing a permanent engagement.

The bandwidth of each module, with a PI-regulated PWM controller, varied for input velocities of 35, 45, and 55 rad/ s, with the maximum 1.51 Hz obtained for 45 rad/ s. This result echoes our previous findings, where Canesi et al. (2017) tested the same device for higher input velocity and found a saturation effect of its bandwidth for higher velocities of the prime mover. This suggests that the limiting dynamic at high frequencies is not given by the rotating speed of the prime mover but by the engaging dynamics of the clutches (Karbasi et al., 2004).

Assuming a sinusoidal motion of the elbow with a peak to peak movement of 90 deg, 1.51 Hz corresponds to a maximum velocity of the joint of 426 deg/s, which is sufficient for everyday tasks such as drinking from a glass (269 deg/s), eating with a spoon (126 deg/s), and pouring from a bottle (92 deg/s), but not for more demanding working tasks such as hammering (842 deg/s) (Buckley et al., 1996).

The maximum load that each module can handle is bounded by the maximum rated torque of the EM clutches. This can be mapped, using Equation (6), to an estimated average torque at the elbow joint of 3.4 N m, which is just above the torque required to keep the average human male forearm in a static posture of 90 deg.

It is worth comparing the performance achieved here with EM clutches with the results reported in Yadmellat et al. (2014), using Magneto-Rheological ones. Yadmellat and colleagues present custom-built clutches, each able to transmit a maximum torque of 15 N m and shown to accurately track a 2 Hz sinusoidal trajectory. Their clutches weigh 2.2 kg each, with a volume of over 650 cm^2^ but, unlike EM cluthces, allow to achieve a smooth and continuous regulation of the output torque, solving the problem of jerky movements typical of discrete systems.

### 4.2. On the Effect on Human Movements

It is increasingly important to evaluate novel wearable assistive technologies based on their effect on the end-user. We believe that this human-in-the-loop validation approach can provide important insights on the limitations and advantages of the innovation, allowing a data-driven design process.

In this work, we compared the performance of of the OTM module with that of a traditional DC motor by looking at its effect on the kinematics and physiology of healthy movements.

Overall, when driven by the OTM module, the assistive device provided fewer benefits in terms of muscular activation and had a more marked effect on movement kinematics than when driven by a traditional DC motor. Specifically, wearing the exosuit powered by the OTM module resulted in more jerky movements, as measured by the SPARC index. This is probably a direct consequence of the discrete nature of the mechanism, driving the assisted joint with small step-like motions along the desired trajectory (see Figure 7 or Figure 8). These fast-changing dynamics are filtered by the compliant transmission between the pulley and the joint but still affect the wearer's movement. We believe that switching to particle-based megneto-rheological clutches, that allow a continuous modulation of the torque being transferred between their input and output shafts, would attenuate or even solve this problem. Previous studies support this proposition (Yadmellat et al., 2014).

Movements assisted by the OTM module resulted in an overall 46.2% reduction in biological torque, vs. an 86.3% reduction achieved with a traditional DC motor, when compared to the unpowered case. This poorer performance of the OTM module is partly caused by: (1) the upper torque limit of the EM clutches, restraining the magnitude of assistance that the actuation can provide; (2) the lower bandwidth of the module, that does not allow it to move fast enough to accommodate the user's intention. This last point is clearly visible in Figure 11D, in red, where the biological torque shows a significant negative peak during the descending phase of the joint (15 s): the user had to push down against the exosuit to extend the elbow.

A corollary of the increase in negative torque, when using the OTM actuation module, is an increase in the activation of the long head of the triceps brachii. Figure 12A shows a peak in the activation of the extensor muscle (bottom plot, in red), of up to 2% of the MVC, during the descending phase of movement (15 s). This peak is present also in the other two conditions, but with much smaller amplitude. Overall, the activity of the triceps brachii increased notably when wearing the exosuit powered by the OTM module, while the biceps brachii was reduced by 30%, when compared to the unpowered condition.

**Figure 13:**
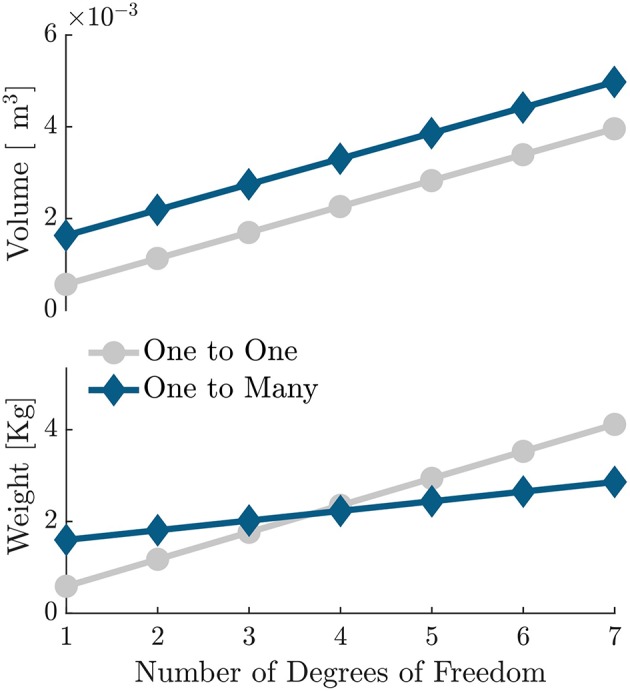
Scalability of the OTM design proposed herein, compared to a traditional one-to-one approach. Volume and weight of the actuation strategy using an OTM paradigm, compared to a setup having similar torque and velocity rating but using one electric motor per DoF.

It is useful to compare the technical characteristics of the proposed OTM system with the traditional approach of using one motor per elbow. In the latter case one would require at least a 70 W motor with an appropriate reduction stage and a motor controller per DoF, which would result in no more than 600 g and 560 cm^3^ per DoF (e.g., Maxon EC-i 40, 70 W, 2-stage planetary gearhead and an ESCON 50/5 module motor driver). Figure 13 shows how the volume and weight of the actuation stage scale with increasing DoF, for a typical one-to-one paradigm and for the OTM strategy proposed here. With the current hardware, the actuation stage would weigh less only for 4 or more DoF. It is worth highlighting, however, that the OTM system only requires one motor controller overall and three simple relay switches per DoF, thus reducing the cost and idle power consumption of the electronics compared to the traditional approach. There is, moreover, ample room for improvement of the size and weight of the OTM modules.

In the present work we explored the potential of an OTM module and compared its performance with a direct drive DC motor, using a wearable device as a test bench for human testing. The DC motor showed higher benefits in assisting human kinematics thanks to its higher bandwidth and smoother motion. However, the OTM paradigm lends itself well to scalable applications, that require independent control of multiple DoFs. This is a common problem in wearable technology, where the complexity of human biomechanics results in an increase in weight, power consumption and control intricacy of the device. If on one side the OTM startegy showed limitations in trajectory tracking, on the other, multiple OTM modules can be driven using a single drive and their control strategy might be optimized reducing the task manifold. In our experiment we used a wearable device for upper limb, and it is known that replicating the dexterity of a human arm is a complex task; we believe that with further improvement on the hardware design, the OTM paradigm has the potential to allow independent control of multiple DoF in a power- and size-effective manner. A different result, for example, might be obtained for well-defined tasks with periodic velocity profiles such as walking, where joint coordination can be achieved by means of synergistic and intermittent assistance on each leg (Asbeck et al., 2015).

## Data Availability

The datasets generated and analyzed for this study are available upon reasonable request.

## Ethics Statement

This study was carried out in accordance with the recommendations of the Institutional Review Board at Nanyang Technological University with written informed consent from all subjects. All subjects gave written informed consent in accordance with the Declaration of Helsinki. The protocol was approved by the Institutional Review Board at Nanyang Technological University.

## Author Contributions

LM conceived the mechanism. MC, MX, EA, and LM designed the OTM module. AK, EA, MX, and LM developed the controller. EA, MX, AK, MC, and LM designed and performed the experiments. AB, AA, and SM helped drafting the paper. All authors analyzed and interpreted the data. All authors provided critical feedback on the manuscript. All authors read and approved the final manuscript.

### Conflict of Interest Statement

EA was employed by company Moveo Walks, Inc., Cambridge, Massachusetts, USA. MC was employed by company Egicon S.R.L., Modena, IT. AK was employed by company Gait Up S.A., Lausanne, Vaud, CH. The remaining authors declare that the research was conducted in the absence of any commercial or finan-cial relationships that could be construed as a potential conflict of interest.

## Footnotes

^1^“Its motion is not measured by another, but all the others are by this, as ten is measured by its half and by its fifth” (Alighieri, 1555).

## Abbreviations

DoF: Degree of freedom
OTP: One-to-many
EM: Electromagnetic
MR: Magneto-rheological
PWM: Pulse width modulation
MJT: Minimum jerk trajectory
EMG: Electromyography
MVC: Maximum voluntary contraction
SPARC: Spectral arc length
RMS: Root mean square
RMSE: Root mean square error.
